# The Insulin-Like Growth Factor 1 Receptor Is Essential for Axonal Regeneration in Adult Central Nervous System Neurons

**DOI:** 10.1371/journal.pone.0054462

**Published:** 2013-01-18

**Authors:** Sebastián Dupraz, Diego Grassi, Diana Karnas, Alvaro F. Nieto Guil, David Hicks, Santiago Quiroga

**Affiliations:** 1 Departamento de Química Biológica, Facultad de Ciencias Químicas, Universidad Nacional de Córdoba y Centro de Investigaciones en Química Biológica de Córdoba (CIQUIBIC), Consejo Nacional de Investigaciones Científicas y Técnicas (CONICET), Córdoba, Argentina; 2 Rhythms, Life and Death in the Retina, Centre National de la Recherche Scientifique (CNRS) UPR-3212 Université de Strasbourg, Institut des Neurosciences Cellulaires et Intégratives, Strasbourg, France; Universidade Federal do ABC, Brazil

## Abstract

Axonal regeneration is an essential condition to re-establish functional neuronal connections in the injured adult central nervous system (CNS), but efficient regrowth of severed axons has proven to be very difficult to achieve. Although significant progress has been made in identifying the intrinsic and extrinsic mechanisms involved, many aspects remain unresolved. Axonal development in embryonic CNS (hippocampus) requires the obligate activation of the insulin-like growth factor 1 receptor (IGF-1R). Based on known similarities between axonal growth in fetal compared to mature CNS, we decided to examine the expression of the IGF-1R, using an antibody to the βgc subunit or a polyclonal anti-peptide antibody directed to the IGF-R (C20), in an *in vitro* model of adult CNS axonal regeneration, namely retinal ganglion cells (RGC) derived from adult rat retinas. Expression of both βgc and the β subunit recognized by C20 antibody were low in freshly isolated adult RGC, but increased significantly after 4 days *in vitro.* As in embryonic axons, βgc was localised to distal regions and leading growth cones in RGC. IGF-1R-βgc co-localised with activated p85 involved in the phosphatidylinositol-3 kinase (PI3K) signaling pathway, upon stimulation with IGF-1. Blocking experiments using either an antibody which neutralises IGF-1R activation, shRNA designed against the IGF-1R sequence, or the PI3K pathway inhibitor LY294002, all significantly reduced axon regeneration from adult RGC *in vitro* (∼40% RGC possessed axons in controls vs 2–8% in the different blocking studies). Finally, co-transfection of RGC with shRNA to silence IGF-1R together with a vector containing a constitutively active form of downstream PI3K (p110), fully restored axonal outgrowth *in vitro*. Hence these data demonstrate that axonal regeneration in adult CNS neurons requires re-expression and activation of IGF-1R, and targeting this system may offer new therapeutic approaches to enhancing axonal regeneration following trauma.

## Introduction

The functional repair of the adult central nervous system (CNS) constitutes a major challenge for modern medicine. Both neurodegenerative diseases and traumatic injuries of the CNS lead to the irreversible loss of cognitive and/or motor functions, associated with permanent disabilities. A prerequisite for functional recovery is axonal and dendritic regeneration in order to re-establish neuronal connections allowing for the normal operation of the CNS. It was formerly thought that axonal regeneration in adult CNS was virtually impossible [1], but the present knowledge of intrinsic [2]–[5] and extrinsic [6]–[8] factors governing axonal regeneration and the formation of new functional synapses has modified this view.

It has been proposed that the mechanisms involved in axonal regeneration of the mature CNS have many features in common with those important in CNS development [9], [10]. However, several aspects of axonal regeneration are still unknown, especially those concerning the regulation of new axon sprouting by extracellular cues. After a lesion, most CNS neurons do not regenerate axons [11], [12]. The regulation of axonal specification and outgrowth in development by extracellular factors is beginning to be understood. Pyramidal cells obtained from embryonic hippocampus have become a recognized model for examining such events in culture. A key observation is that a particularly early event in neurons that do not yet exhibit a discernible axon (defined as stage 2 of differentiation [13]), is the segregation of activatable IGF-1 receptors (IGF-1R) to one single neurite. Subsequently, phosphatidylinositol-3 kinase (PI3K) and its product PIP_3,_ accumulate in the distal region and growth cone of that same neurite, together with the IGF-1R. These events are critical for the outgrowth of the future axon [14]–[16]. Indeed, designation of the axon requires activation of PI3K by IGF-1R [13], [14]. A similar system is involved in regeneration of the adult peripheral nervous system [17], [18]. In contrast there is no published information about the possible participation of the IGF-1R-PI3K pathway in axonal regeneration of adult mammalian CNS neurons.

To address this question, as an experimental model we used retinal ganglion cells (RGC), since these have been shown to survive and regenerate neurites *in vitro* [19] and both IGF-1 and IGF-1R are widely present in different retinal cells in mammals including man [20], [21]. Our results demonstrate that the expression of IGF-1R is significantly higher in cultured adult RGC than in recently disaggregated adult retina cells and that the βgc containing variant of the IGF-1R is enriched at the third distal axon. Loss of function experiments using a blocking IGF-1R antibody or a shRNA directed against IGF-1R, as well as pharmacological inhibition of PI3K signaling, repressed axonal regeneration. Co-transfection of IGF-1R-suppressed cells with a constitutively active form of PI3K rescued the phenotype with the outgrowth of one or more axons.

## Materials and Methods

### Animals

All animal procedures adhered strictly to the guidelines of the Association for Research in Vision and Ophthalmology Statement on the Use of Animals in Ophthalmic and Visual Research, and were performed using approved protocols by the Board of Animal Welfare, School of Chemical Sciences, National University of Cordoba, Argentina.

### Primary Antibodies

The following primary antibodies were used: mouse monoclonal antibody to c-myc (Sigma) diluted 1∶600; mouse monoclonal antibody to the axonal marker Tau-1 (Calbiochem) diluted 1∶600; rabbit polyclonal antibody to βgc [22] 1∶50 for immunofluorescence and 1∶250 for Western blots; rabbit polyclonal antibody to the neurofilament 200 kD sub-unit (NF200) (Sigma) diluted 1∶600 (NF200 is expressed by RGC and horizontal cells in the retina: [23]); rabbit monoclonal antibody to Phospho-IGF-1 Receptor (tyr980-C14A11-Cell Signaling) diluted 1∶50; goat polyclonal antibody to IGF-1r β subunit (20C sc-713-G, Santa Cruz Biotechnology) diluted 1∶250 for Western blots and a rabbit monoclonal antibody to phospho p85 (tyr458)/p55(Tyr199) (Cell Signaling) diluted 1∶200.

### Cell Culture

Retinal cultures were prepared essentially as previously described [19], [24], [25]. Adult Wistar rats (weighting more than 200 g) were anesthetized with CO_2,_ killed by cervical dislocation and the eye tissue was rapidly removed. Retinas were isolated into fresh CO_2_-independent Dulbecco’s Modified Eagle’s Medium (DMEM-CO_2_) (Gibco) by circumferential section of the cornea and removal of the anterior chamber. Major blood vessels were excised, and retinas were then chopped into small fragments, washed in Ringer’s solution without Ca^2+^ or Mg^2+^, supplemented with 0.1 mM EDTA, and incubated in 0.5 ml 0.2% activated papain (Sigma Aldrich Life Sci., Saint Quentin Fallavier, France) in the same buffer for 30 minutes at 37°C. The tissue was dissociated by repeated gentle trituration. To enrich the suspension in RGC, we modified a protocol originally developed for chicken retina [26]: cells were spun for 5 min at 100 g using a bovine serum albumin (BSA) discontinuous gradient (2.5%–4%–5%). Screening of fractions for NF200 expression showed about 40% immunopositive cells in the fraction at the 2.5%–4% interface (data not shown). This fraction was collected, diluted and seeded in Neurobasal A medium containing B27 supplement (Gibco) plus 10% bovine fetal serum and penicillin-streptomycin (10 IU/l), into 24-well tissue culture plates containing coverslips previously coated with poly-l-lysine (2 µg/cm^2^ for 2 hours) followed by laminin (1 µg/cm^2^ overnight; both from Sigma Aldrich Life Sciences. Seeding was performed at an initial density of 2.5 or 5 × 10^5^ cells/cm^2^, and cells were incubated at 37°C in a humidified atmosphere of 5% CO_2_-95% air. After 24 h the culture medium was changed to Neurobasal A medium containing the B27 supplement without serum.

### Cell Transfection

By screening fetal rat brain expression library with an anti-βgc antibody [22] we cloned a 150-bp sequence that is essentially identical to a segment of the published IGF-1R. The cloned sequence encodes a peptide close to the extracellular amino terminus of the IGF-1R β subunit. Because there is only one known IGF-1R gene and the different receptor variants should be generated by alternative splicing, we used a slightly modified published siRNA sequence to suppress IGF-1R synthesis (GCCCATGTGTGAGAAGACC; [13], [27]). A scrambled DNA target sequence (GAACGGTCGCAGTGTACCA) was created using the siRNA Wizard™, InvivoGen. cDNAs encoding shRNAs were inserted in a discistronic vector pSuper.neo+GFP (pSuper RNAi System-OligoEngine) under the control of the H1 RNAIII polymerase promoter, and the transfection marker GFP was under the control of the PGK promoter. The resulting plasmids were referred to as IGF-1R shRNA and scrambled sequence RNA (ssRNA). The myc tagged, constitutively active p110 construct was a generous gift from Dr. L. Williams [28]. The plasmids were mixed with Lipofectamine 2000 and added to the neurons 12 h after plating. Transfection efficiencies were from 10 to 20%.

### Immunofluorescence Microscopy

Cells were fixed for 1 h at room temperature with 4% (wt/vol) paraformaldehyde in phosphate buffered saline (PBS), pH 7.4, containing 4% (wt/vol) sucrose. Cultures were washed with PBS, permeabilized with 0.1% (vol/vol) Triton X-100 in PBS for 6 min, and again washed in PBS. After labeling with a first primary antibody (1–3 h at room temperature) and washing with PBS, cultures were incubated with fluorescent secondary antibody (conjugated to Alexa fluor 488, 546 or 633; 1 h at 37°C) and washed with PBS. The same procedure was repeated for the second and third primary and secondary antibodies. The cells were observed with a Zeiss Pascal 5 confocal microscope or an Olympus FV-1000 confocal spectral microscope. Images were captured and digitized using LSM Image software. All images were printed using Adobe PhotoShop, with digital enhancement performed equally for all panels.

### Gel Electrophoresis and Western Blot

Proteins were separated by SDS-polyacrylamide gel electrophoresis. The concentration of acrylamide of the resolving gel was 11%. The resolved proteins were transferred to polyvinylidene difluoride (PVDF) membranes in Tris-glycine buffer containing 20% methanol. The membranes were first dried, washed with Tris-buffered saline (TBS; 10 mM Tris pH 7.5, 150 mM NaCl) and then blocked, or directly blocked for 1 h in TBS containing 5% BSA. The blots were incubated with the primary antibodies in PBS containing 0.05% Tween 20, for 2 h at room temperature. After washing with TBS containing 0.05% Tween 20, the membranes were incubated with goat or donkey anti-rabbit IgG DyLight 800 fluorescent dye-conjugated secondary antibody (Pierce Biotechnology) for 1 h at room temperature (dilution 1∶15000). After washing with TBS containing 0.05% Tween 20 the blots were analyzed using a Li-COR Odyssey Infrared Imaging System (LI-COR Biosciences) according to the manufactureŕs instruction.

### Statistical Analysis

All experiments were replicated at least 3x with independent samples and data are expressed as mean values ± S.E. Statistical analysis was performed using a two-tailed unpaired Student’s t test with Excel. Differences were considered significant for *p*<0.05.

## Results

**Figure 1:**
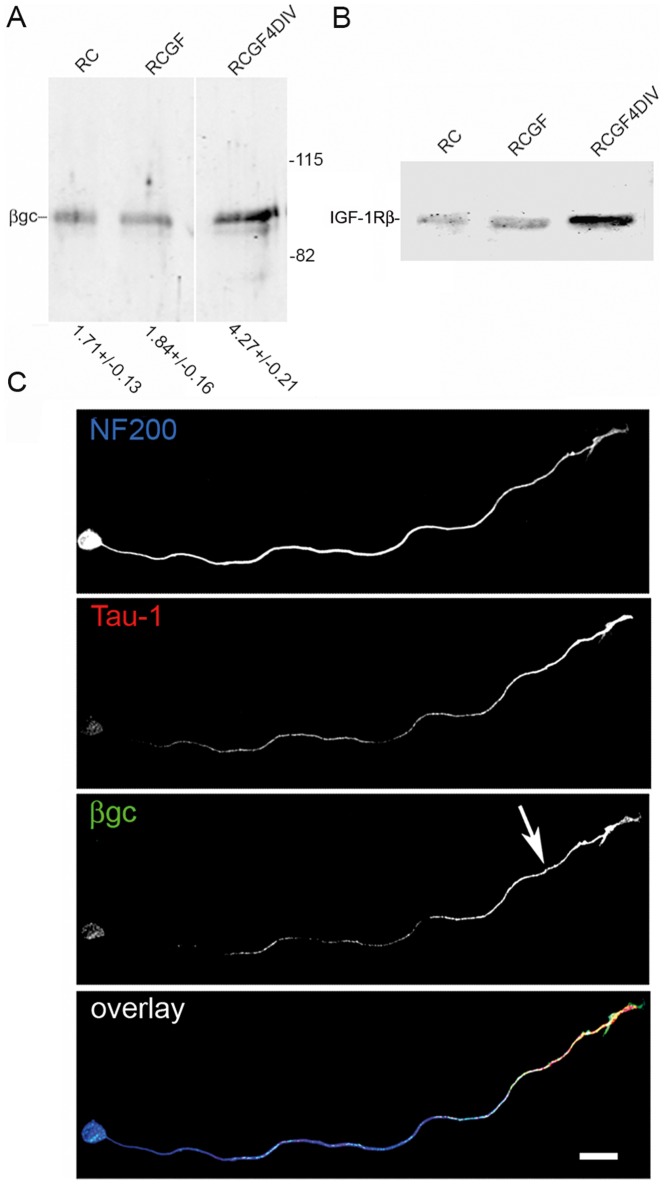
The IGF-1R β subunit is enriched at the distal-most third of the axon in adult RGC regenerating in culture. A) Western blot of protein from total retina disaggregated cells (RC), a fraction enriched in RGC (RGCF-see methods) and the same fraction after 4 days in culture (RGCF 4DIV) detected using an anti-βgc antibody. Equal amounts of protein from the three different fractions were loaded onto each lane. Note the increased intensity of βgc staining in the RGC 4DIV fraction. Numbers below are the O.D. (arbitrary units) +/− s.e.m. n = 3 independent experiments. B) Western blot prepared as in Fig. 1 A developed using a polyclonal antibody to the IGF-1R β subunit (C20-Santa Cruz Biotechnology). Note the increased intensity of C20 staining in the RGC 4DIV fraction. C) Triple immunofluorescence micrographs of a rat adult RGC regenerating in culture after 3 DIV showing the distribution of the RGC marker NF200 (blue, top panel), the axonal marker Tau-1 (red, second panel) and the β subunit of the IGF-1R (βgc) (green, third panel) in adult RGC neurons regenerating in culture. The lower panel shows a merged image of the three stainings. Note the enrichment of βgc at the distal end of the axon (arrow). Calibration bar = 20 µm.

**Figure 2:**
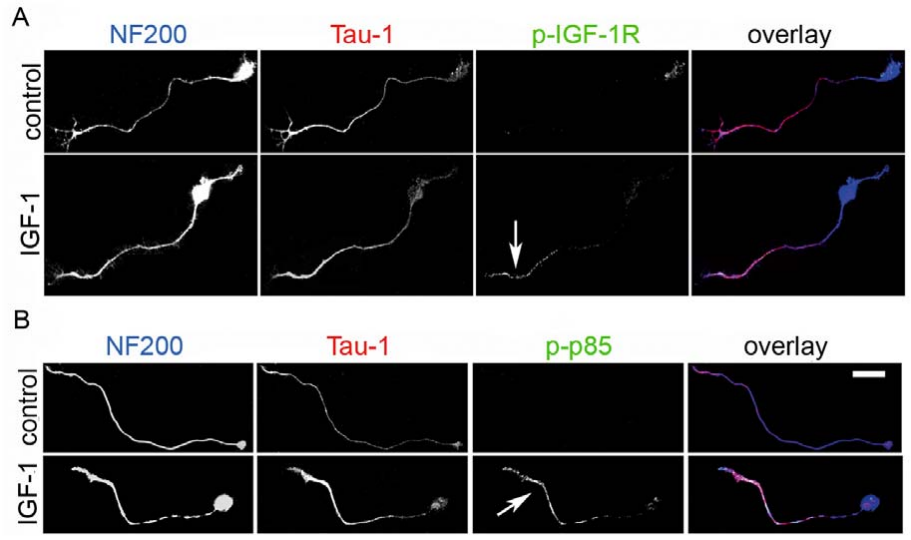
Stimulation with IGF-1 triggers the polarized activation of the IGF-1R and PI3K in the axons of adult rat RGC regenerating in culture. A) Triple immunofluorescence micrographs of a rat adult RGC regenerating in culture after 3 DIV showing the distribution of the RGC marker NF200 (blue), the axonal marker Tau-1 (red) and the phosphorylated (active) form of the IGF-1R (green, pIGF-1R). Cells were cultured for 3 days, deprived of insulin for 4 h and stimulated for 5 min in control medium or medium containing 10 nM IGF-1. Note the significant activation of the IGF-1R, especially at the distal third of the axon (arrow). B) Triple immunofluorescence micrographs of a rat adult RGC regenerating in culture after 3 DIV showing the distribution of the RGC marker NF200 (blue), the axonal marker Tau-1 (red) and the phosphorylated form of the PI3k regulatory subunit p85 (green, p-p85). Cells were treated as described above (Fig. 1A). Note the pronounced activation of PI3K, especially within the distal axon (arrow). Calibration bar = 20 µm.

**Figure 3:**
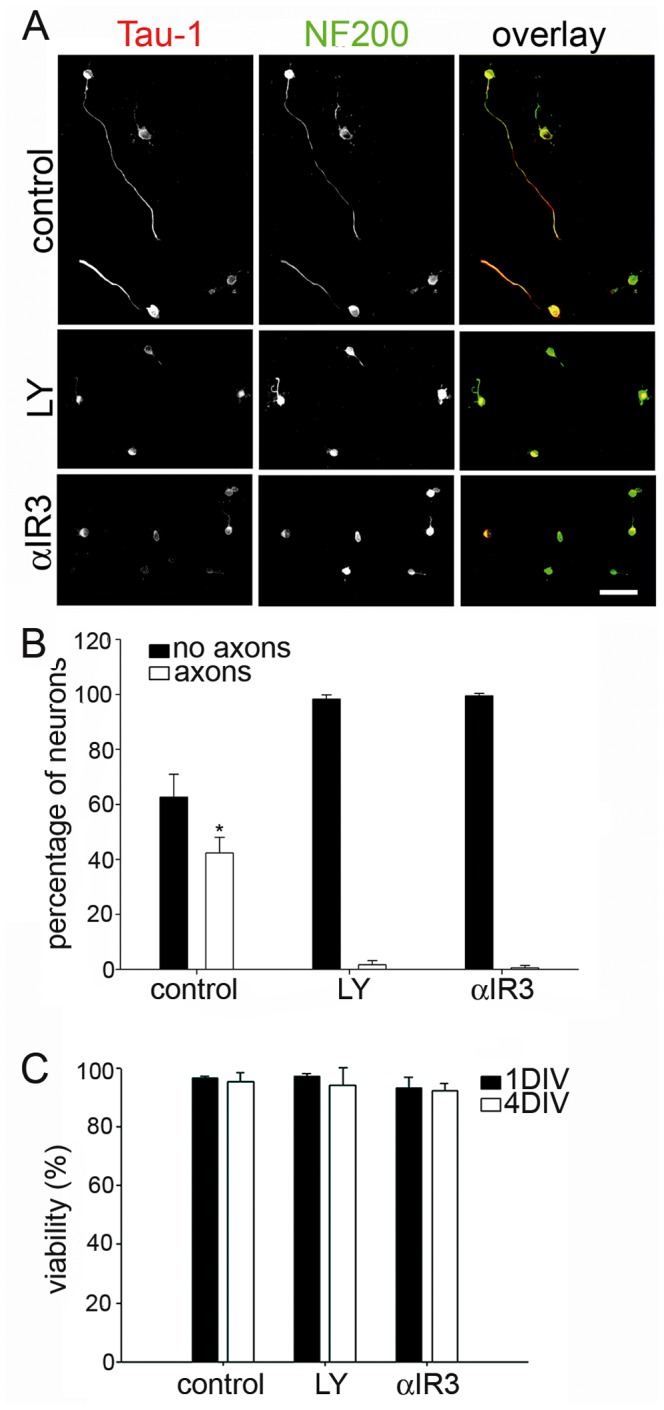
Treatment with the PI3K inhibitor LY 294002 or the IGF-1R blocking antibody αIR3 prevents axon formation in adult RGC in culture. A) Double immunofluorescence showing the distribution of the RGC marker NF200 and the axonal marker Tau-1 in cultured adult RGC. Cells were cultured for 3 days in control conditions or in the presence of 20 nM LY 294002 (LY) or αIR3 (10 mg/ml). Calibration bar = 40 µm. B) Percentage (+/− s.e.m.) of adult RGC cultured for 3 days in the different conditions described above (Fig 3A), exhibiting or lacking axons. n = 3 independent experiments. At least 100 cells were scored for each condition. * Significantly different from LY and a IR3 p≤0,0005. C) Cell viability (determined by the criterion of propidium iodide exclusion [52]) after 1 or 4 DIV. n = 3 independent experiments. At least 100 neurons were scored for each condition.

**Figure 4:**
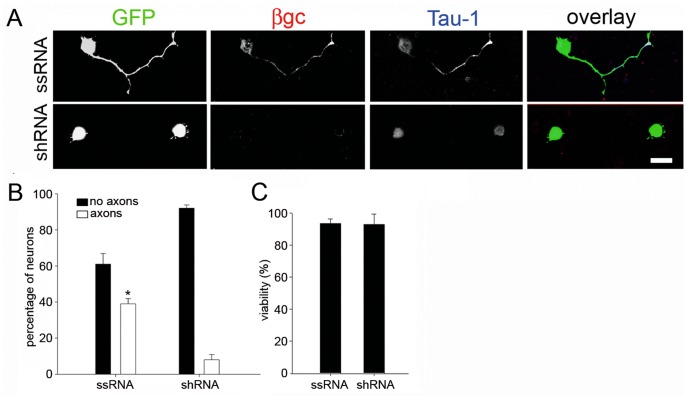
Silencing IGF-1R expression prevents axonal formation in adult RGC in culture. A) Double immunofluorescence of adult RGC after 3 days in culture transfected with scrambled sequence RNA (ssRNA) or shRNA directed to the IGF-1R (shRNA) showing the distribution of the IGF-1R β subunit and the axonal marker Tau-1. GFP shows the efficiency of cell transfection. Calibration bar = 10 µm. B) Percentage (+/− s.e.m.) of adult RGC treated as described above (Fig. 4A) exhibiting or lacking axons. n = 3 independent experiments. At least 100 cells were scored for each condition. * Significantly different from shRNA p≤0,0005. C) Cell viability (determined by the criterion of propidium iodide exclusion [52]) after 4 DIV. n = 3 independent experiments. At least 50 neurons were scored for each condition.

**Figure 5:**
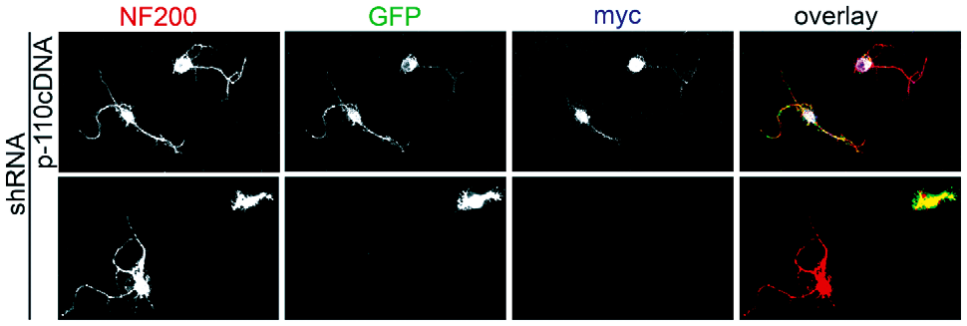
Co-transfection of IGF-1R-suppressed adult RGC with a constitutively active form of the PI3k catalytic subunit p-110 restores the ability of the neurons to form axons. Double immunofluorescence of adult RGC showing the distribution of NF200 (displaying neuronal shape) and myc (marker of transfection with cDNA encoding for a constitutively active form of the PI3K regulatory subunit p110). GFP is a marker of transfection efficiency with the shRNA directed to IGF-1R. Cells were transfected and cultured for 4 days. Calibration bar = 10 µm.

[LOOSESR]We first investigated the expression and sub-cellular distribution of IGF-1R in adult rat RGC after 3 days in culture. We found that the expression of βgc containing-IGF-1R was relatively low in both total disaggregated adult retina cells and a fraction enriched in RGC. In contrast, RGC expressed significantly higher amounts of βgc after 4 days in culture (Fig. 1A). A similar result was obtained using a polyclonal antipeptide antibody to the β subunit of the IGF-1R (C20-Santa Cruz Biotechnology-Fig. 1.B). Immunofluorescence experiments showed that βgc was highly enriched at the distal-most third of axons, and the growth cone of adult RGC in culture (Fig. 1C). We next investigated the expression and distribution of phosphorylated (active) IGF-1R and the phosphorylated form of the PI3K regulatory sub-unit p85 in adult rat RGC grown for 3 days in the presence of high insulin (sufficient to activate both the insulin receptor and IGF-1R), then deprived of growth factors for four hours and challenged with 10 nM IGF-1 for 5 min. We observed the expression of significant amounts of both active IGF-1R (Fig. 2A) and the phosphorylated form of p85 enriched within the distal-most third of the axon and the growth cone in RGC. To study the likely relationship between polarized activation of the IGF-1R and the stimulation of axonal regeneration in RGC, we performed loss-of-function experiments by three different approaches. Addition of an IGF-1R blocking antibody prevented axonal outgrowth in the vast majority of neurons (Fig. 3a, bottom): whereas 40% of control neurons developed axons, only ∼2% of neurons treated with blocking antibody exhibited axons (Fig. 3b). Secondly, using a shRNA derived from the IGF-1R sequence which uniformly silences the expression of βgc-containing IGF-1R [13] we observed that most of the silenced cells challenged with IGF-1 failed to form axons (Fig. 4a). For quantitative measures, we scored the amount of neurons exhibiting axons in cultures transfected with a scrambled sequence RNA (ssRNA) or with IGF-1R-targeted shRNA. Quantification showed that only about 8% of shRNA-transfected neurons formed a discernible axon compared to 42% of controls containing ssRNA (Fig. 4b). Finally, most cells cultured in the presence of the PI3K inhibitor LY294002 also failed to form axons (Fig. 3a middle). As shown in Fig. 3b, less that 3% treated neurons exhibited axons after 3 days in culture, in contrast to neurons cultured in the absence of the PI3K inhibitor in which almost 40% cells exhibited axons. Reduced polarization of RGC after treatments with αIR3, LY294002 or after shRNA addition were not related to failing neuronal health because cell viability was not significantly affected by any of those treatments (Fig. 3 C and 4 C) Since PI3K operates downstream of the IGF-1R, and to further eliminate possible artefacts due to toxic or deleterious effects of transfection with the IGF-1R shRNA, we co-transfected cells with IGF-1R shRNA and with a cDNA encoding a myc-tagged constitutively active form of PI3K, p110. The results showed that co-transfection completely rescued the phenotype, with over 90% of co-transfected cells exhibiting axon-like processes compared to 8% of cells transfected with IGF-1R shRNA alone (Fig. 5).

## Discussion

The data presented here indicate that one of the earliest events in regeneration of adult CNS axons, exactly as in embryonic CNS neurons, is the expression and functioning of the IGF-1R, including the βgc containing variant. βgc is an immunochemically distinct β subunit of IGF-1R [13], [22], highly enriched in the distal-most third of the axon and growth cones during neuronal differentiation ([22], [29]-For a detailed molecular description of this subunit see Materials and Methods under Cell Transfection). Within the developing hippocampus, passage from stage 2 of differentiation (prior to the appearance of a discernible axon) to stage 3 (appearance of an identifiable axon) is marked by the segregation of activatable IGF-1R to one neurite with the subsequent activation of PI3K, axonal specification and outgrowth [13]. Previous results from our laboratory showed that the sequence encoding the βgc subunit is contained within that of the IGF-1R [13]. Thus, the βgc variant must be the result of alternative splicing of the single *igf-1* receptor gene known to exist in rats.

Several studies have demonstrated the involvement of IGF-1R and downstream signaling pathways, including PI3K and cdc42, in the regulation of axonal specification and outgrowth during differentiation in at least two types of central neurons: hippocampal pyramidal neurons [13] and corticospinal motor neurons [30]. This receptor system is also involved in axonal regeneration in adult peripheral nervous system [17], [18]. In fishes, adult CNS neurons are able to regenerate throughout the entire life span, and the IGF-1/PI3K system is also required [31]. The possible role of this receptor/signaling pathway in the regeneration of axons in adult central neurons has not yet been studied. In order to perform these studies we chose RGCs as a model for mature neurons for the following reasons: i) the axons of these cells, located in the optic nerve, are affected in many disorders including traumatic optic neuropathy [32], ischemic optic neuropathy [33], optic neuritis [34] and glaucoma [35]. Functional recovery after these insults requires overcoming several barriers compromising RGC axonal outgrowth potential; ii) all the retinal neurons, including RGC, are able to survive and develop neurites in primary cultures including those from adult animals and humans [19], [23], [36]; iii) retinal cells in culture have been extensively used to study the cellular and molecular mechanisms involved in axonal regeneration [37], [38]; iv) retina cells express IGF and IGF-1R widely [20], [21]; and v) the optic nerve is a readily accessible region of the CNS in which to further explore these studies *in vivo*.

The expression of IGF-1R is developmentally regulated in brain, reaching highest levels at embryonic and early postnatal stages [39], [40]. Expression of IGF-1R is relatively low in the adult retina. We observed a significant increase in the expression of this receptor after disaggregation and culture of adult RGC. These results suggest that axon severing during disaggregation can trigger IGF-1R re-expression as part of a “regeneration program”. The fact that polarized activation of the IGF-1R at the growth cone and distal axon is sufficient to stimulate axonal regeneration in the absence of any other growth factor is a novel observation. This is somewhat in contradiction to the observation that CNS neurons, using RGC as an experimental model, require a cocktail of trophic factors in order to survive and regenerate neurites *in vitro* [41]. We cannot rule out the secretion of additional trophic factors by surrounding retinal cells in our culture model, but the loss-of-function experiments using blocking IGF-1R antibody or shRNA targeting IGF-1R demonstrate that the activation of IGF-1R is essential for renewed axonal outgrowth in adult RGC. Previous studies have shown that IGF-1 protects axotomized RGC from secondary death via PI3K dependent akt phosphorylation and activation of caspase-3 *in vivo* [42]. IGF-1, IGF-1R and IGF binding proteins, which modulate ligand availability and receptor activation, are all found within the mammalian retina including man [21]. It is of interest in the present context that IGF-1 mRNA is localized to RGC cell bodies, whereas IGF-1R are more widespread [20]. There is a notable down-regulation of endogenous IGF-1 expression by the RGC during postnatal maturation [43], which may be one of the reasons of the very limited axonal regeneration in adult optic nerve. In keeping with this idea, IGF-1 delivery into postnatal rat eyes partly rescues RGC from programmed cell death and maintains higher numbers of axons within the optic nerve [44]. And IGF-1 is down-regulated rapidly in RGC following optic nerve transection, leading to interruption in PI3K signaling and RGC death [45]. Taken together with the data presented here, this strongly suggests that the IGF-1/IGF1-R/PI3K system is important for regulating both cell survival and axonal regeneration in adult RGC.

Several neurotrophic factors have been implicated in ameliorating survival and axonal regeneration in adult RGC. Hepatocyte growth factor improves both RGC survival and axonal regeneration *in vivo* and *in vitro*, and also exerts its effects through activation of Akt [46]. Similarly, BDNF administration to adult RGC *in vitro* stimulates neurite outgrowth through the PI3K pathway [47]. Fibroblast growth factors (FGFs) probably play multiple roles in RGC survival and process growth, since *in vitro* studies showed that FGF-2 and FGF-9 stimulate adult RGC survival without affecting axon extension [24] while studies *in vivo* demonstrated that stimulation of axon growth in adult RGC by FGF-2 depends on the activation of the extracellular signal-regulated kinase 1/2 but not PI3K [48], [49]. Although there are numerous similarities between axon outgrowth in embryonic and adult CNS, there are also some differences. While BDNF (acting through the PI3K pathway) has been shown to be involved in adult RGC process regeneration [47], this factor does not trigger axonal specification and outgrowth in embryonic hippocampal neurons [13] or cortical motor neurons [30].

The results shown here indicate that PI3K activation is necessary for the activated IGF-1R induction of RGC axonal regeneration. Moreover, transfection with a constitutively active form of PI3K was sufficient to trigger axonal regeneration in the absence of any other extracellular stimulus. It has also been shown that PI3K activation significantly increases axonal regeneration over the level induced by pten (phosphatase and tensin homolog) deletion [50]. This could be very interesting in the field of gene therapy directed towards promotion of re-growth of damaged RGC axons in several neurodegenerative conditions. This is especially cogent given that up-regulation of IGF-1 within the eye leads to appearance of diabetes-like alterations [51], which cautions against direct use of this growth factor in promoting axonal repair.
